# Real-world outcomes of encorafenib, cetuximab ± binimetinib for *BRAF*‑mutated metastatic colorectal cancer: the BEETS (JACCRO CC‑18) study

**DOI:** 10.1093/oncolo/oyag068

**Published:** 2026-02-27

**Authors:** Daisuke Kotani, Eisuke Inoue, Tadamichi Denda, Chiaki Inagaki, Tomomi Kashiwada, Yoshiaki Mihara, Akinori Sugaya, Yusuke Suwa, Takashi Ohta, Hidekazu Kuramochi, Kotoe Oshima, Satoshi Yuki, Manabu Shiozawa, Akihito Tsuji, Kei Muro, Wataru Ichikawa, Masashi Fujii, Yu Sunakawa

**Affiliations:** Department of Gastroenterology and Gastrointestinal Oncology, National Cancer Center Hospital East, Kashiwa, 277-8577, Japan; Showa Medical University Research Administration Center, Showa Medical University, Tokyo, 142-8555, Japan; Division of Gastroenterology, Chiba Cancer Center, Chiba, 260-8717, Japan; Department of Medical Oncology, Kindai University Faculty of Medicine, Sakai, 590-0197, Japan; Department of Medical Oncology, Saga-Ken Medical Center Koseikan, Saga, 840-8571, Japan; Division of Medical Oncology, Showa Medical University Fujigaoka Hospital, Yokohama, 227-8501, Japan; Department of Medical Oncology, Ibaraki Prefectural Central Hospital, Kasama, 309-1793, Japan; Department of Surgery, Gastroenterological Center, Yokohama City University Medical Center, Yokohama, 232-0024, Japan; Department of Clinical Oncology, Kansai Rosai Hospital, Amagasaki, 660-0064, Japan; Department of Medical Oncology, NTT Medical Center Tokyo, Tokyo, 141-8625, Japan; Division of Gastrointestinal Oncology, Shizuoka Cancer Center, Sunto-Gun, 411-8777, Japan; Department of Gastroenterology and Hepatology, Hokkaido University Hospital, Sapporo, 060-8648, Japan; Department of Surgery, Kanagawa Cancer Center, Yokohama, 241-8515, Japan; Department of Clinical Oncology, Faculty of Medicine, Kagawa University, Kagawa, 761-0793, Japan; Department of Clinical Oncology, Aichi Cancer Center Hospital, Nagoya, 464-8681, Japan; Division of Medical Oncology, Showa Medical University Fujigaoka Hospital, Yokohama, 227-8501, Japan; Japan Clinical Cancer Research Organization (JACCRO), Tokyo, 101-0051, Japan; Department of Clinical Oncology, St. Marianna University School of Medicine, Kawasaki, 216-8511, Japan

**Keywords:** BRAF mutated, metastatic colorectal cancer, cetuximab, encorafenib, binimetinib

## Abstract

**Background:**

Triplet therapy (encorafenib, cetuximab, and binimetinib) is recommended in Japan for *BRAF* V600E mutated metastatic colorectal cancer (mCRC) patients with poor prognostic factors (PFs) based on subgroup analyses of the BEACON CRC trial. We, therefore, conducted a nationwide prospective observational study to evaluate the real-world effectiveness of triplet versus doublet therapy (encorafenib plus cetuximab).

**Patients and Methods:**

The BEETS trial (UMIN000045530) enrolled *BRAF*-mutated mCRC patients who received triplet or doublet as second- or third-line treatment. The primary endpoint was overall survival (OS). Exploratory analyses using inverse probability weighting (IPW) based on propensity scores were performed to adjust for poor PFs (ECOG performance status ≥1, ≥3 metastatic sites, elevated C-reactive protein, or unresected primary tumor).

**Results:**

In 203 enrolled patients, 195 patients were evaluable. Median age was 67 years; 52% were male. Dose intensity for encorafenib and cetuximab was comparable between the triplet and doublet cohorts. In the overall cohort, median OS and progression-free survival (PFS) were 12.9 and 4.9 months, respectively. After IPW adjustment, median OS was 14.0 months in the triplet cohort and 12.9 months in the doublet cohort (HR 0.87, 95%CI 0.57-1.33). Median PFS was 5.3 versus 4.2 months (HR 0.75, 95%CI 0.48-1.19). Among patients with at least one poor PF, both OS and PFS numerically favored the triplet regimen.

**Conclusions:**

In real-world clinical practice, triplet and doublet therapies showed comparable survival outcomes, consistent with the BEACON trial. Triplet therapy may provide potential clinical benefit in patients with poor PFs.

Implications for Practice Encorafenib plus cetuximab with or without binimetinib is clinically effective for patients with BRAF-mutated metastatic colorectal cancer in clinical practice. Three drugs, triplet therapy showed a trend toward improved survival in patients with poor prognostic factors, suggesting a potential role for risk-adapted regimen selection.

## Introduction

*BRAF* mutations occur in approximately 8%-12% of metastatic colorectal cancer (mCRC), with the vast majority harboring the *BRAF* V600E substitution.^1^ Patients with *BRAF* V600E-mutated mCRC exhibit aggressive tumor biology and markedly poor prognosis, with median overall survival (OS) typically less than 15 months under conventional chemotherapy-based regimens.^2^ The pivotal phase III BEACON CRC trial evaluated encorafenib (BRAF inhibitor) plus cetuximab (anti-EGFR antibody) with or without binimetinib (MEK inhibitor) in previously treated *BRAF* V600E-mutated mCRC. Both doublet and triplet regimens significantly improved OS and objective response rate (ORR) compared with irinotecan-based chemotherapy plus cetuximab. Although the trial established these targeted combinations as new standards of care, the absolute survival gain remained limited (median OS ∼9 months), and most patients experienced disease progression within 6 months.^3^ Moreover, subgroup analyses and exploratory biomarker studies indicated molecular heterogeneity within *BRAF* V600E mutant tumors—suggesting that treatment benefit may differ depending on transcriptional subtype, tumor burden, or co-alterations in the WNT, RTK, or PI3K pathways.^4^

In Japan, both the encorafenib–cetuximab doublet and the encorafenib–binimetinib–cetuximab triplet are approved and reimbursed for use in second- or third-line settings. The Japanese Colorectal Cancer treatment guidelines recommend triplet therapy particularly for patients with adverse prognostic factors such as high tumor burden, multiple metastatic organs, or elevated inflammatory markers (e.g., C-reactive protein >1 mg/dL), based on the results of subgroup analysis in the BEACON CRC trial.^5^ However, clinical evidence to support this stratified recommendation has been limited, especially in real-world Asian populations. Western real-world data, including the CONFIDENCE and CAPSTAN studies,^6^^,^^7^ have provided early reassurance of efficacy and safety outside clinical trials, but Asian prospective datasets remain scarce.

The BEETS (JACCRO CC-18) study was, therefore, established as a nationwide, prospective, observational, and translational research project to address these evidence gaps. Through this approach, BEETS aims not only to clarify the comparative clinical utility of triplet versus doublet targeted therapy in a real-world Asian setting but also to elucidate the molecular underpinnings of treatment response, resistance, and disease heterogeneity in *BRAF*-mutated mCRC.^8^

## Methods

### Study design

The BEETS (JACCRO CC-18) trial was a nationwide, multicenter, prospective observational and translational study designed to evaluate the real-world efficacy and safety of combination targeted therapies for *BRAF*-mutated mCRC. We also planned to identify clinical factors that could be used to guide treatment selection by comparing clinical outcomes between patients treated with triplet therapy and those treated with doublet therapy. The study was organized by the Japan Clinical Cancer Research Organization (JACCRO) and conducted across 66 participating institutions in Japan. Eligible patients had histologically confirmed colorectal adenocarcinoma harboring any *BRAF* mutation by tissue genomic testing, were ≥20 years, ECOG PS 0-2, and had measurable/evaluable disease per RECIST v1.1. Key exclusions included interstitial lung disease, recent cardiovascular events, active infections, and other serious comorbidities.^8^ All patients provided written informed consent; the protocol was approved by the St. Marianna University ethics committee (approval no. 5413) and adhered to the Declaration of Helsinki.

In addition, we conducted blood-based biomarker research to identify novel biomarkers predictive of treatment efficacy, optimize treatment selection, and explore the mechanisms of resistance to BRAF inhibitor combination therapies.

### Study end points

The primary endpoint of this study was OS. Secondary endpoints included ORR, disease control rate (DCR), tumor regression rate (TRR), time to response (TTR), duration of response (DoR), PFS, and safety. Patients who remain alive or are lost to follow-up will be censored as of the date of last confirmation of survival. ORR represents the proportion of patients showing an overall response of complete response (CR) or partial response (PR) for measurable lesions. Disease control rate (DCR) is defined as the proportion of patients with an overall response of CR, PR, or stable disease for measurable lesions. TRR is calculated based on the difference in the sum of diameters of target lesions measured according to RECIST criteria, version 1.1 between before and during BRAF inhibitor combination treatment. PFS is the period from enrollment to date of disease progression or the date of death from any cause, whichever is earlier. If surgery is performed based on the consideration that surgical removal should be applied to the primary lesion or a metastatic lesion, relapse after surgery will be deemed as progressive disease (PD). The frequency of the worst grade in all courses will be calculated for each adverse event according to Common Terminology Criteria for Adverse Events (CTCAE) version 5.0.

### Treatment

Patients received either the triplet regimen (encorafenib + binimetinib + cetuximab) or the doublet regimen (encorafenib + cetuximab). Treatment choice was determined by each investigator to reflect actual clinical practice rather than random assignment. Recommended doses followed Japanese labeling at study initiation. Treatment continued until disease progression, unacceptable toxicity, or withdrawal of consent. Dose interruptions and reductions were permitted per standard clinical guidelines. Concomitant medications, including corticosteroids or anti-diarrheal agents, were allowed as clinically indicated.

### Statistical design

In the BEACON CRC trial, the median survival times (MSTs) in the triplet therapy arm, doublet therapy arm, and control group were 9.0 months, 8.4 months, and 5.4 months, respectively. Considering that the present study evaluates outcomes under real-world clinical practice, the MST of the study population was conservatively estimated to be 8.0 months. This value was compared with an MST of 5.4 months as the reference. To achieve a power of 0.9 with a two-sided test at a significance level of 0.05, a total of 186 patients is required, assuming an accrual period of 2 years and a follow-up period of 2 years. Allowing for a small proportion of patients to be excluded from the analysis, the target sample size was set at 200 patients.

The efficacy analysis set consisted of all study participants who were enrolled in the study and for whom at least one post-enrollment assessment of the study outcomes was performed. The safety analysis set was defined as all patients receiving at least one dose of study treatment. Survival functions for time-to-event outcomes were estimated using the Kaplan–Meier method, and hazard ratios (HRs) with 95% confidence intervals (CIs) were estimated using Cox models. For each cohort, the median OS was compared with a prespecified reference value (*m*_0_ = 5.4) using an interval-inversion approach: the null hypothesis *H*_0_: median OS = *m*_0_ was rejected at the two-sided 0.05 level if *m*_0_ lay outside the Brookmeyer–Crowley 95% confidence interval for the median.

Comparative analyses between triplet and doublet cohorts were conducted exploratorily, without prespecified formal hypothesis testing. To address potential selection bias arising from nonrandomized treatment assignment, inverse probability weighting (IPW) based on propensity scores was applied. Propensity scores were estimated using the covariate balancing propensity score (CBPS) method, incorporating baseline covariates reflecting poor prognostic factors: ECOG performance status (PS) (0 vs ≥1), number of metastatic organs (<3 vs ≥3), CRP level (<1 mg/dL vs ≥ 1 mg/dL), and primary-tumor status (resected vs unresected). Weights corresponding to the average treatment effect were calculated and truncated at the upper 5th percentile to reduce the influence of extreme weights. Covariate balance after weighting was assessed using standardized mean differences, with values less than 0.1 considered indicative of adequate balance. Weighted Cox proportional hazards models with robust variance estimators were used to estimate HRs for OS and PFS, and subgroup analyses were conducted for patients with one or more poor prognostic factors.

All statistical analyses were executed using R version 4.5.

### Data management

Data were collected via an electronic data capture system maintained by the JACCRO Data Center. On-site and remote monitoring ensured data accuracy and protocol compliance. Investigators could withdraw patients for adverse events, clinical deterioration, or at patient request. All data cut-off analyses were predefined in the statistical analysis plan (SAP) prior to database lock (February 2025).

## Results

### Patient disposition and baseline characteristics

From October 2021 to November 2023, 203 patients were enrolled at 66 institutions; 195 received at least one dose and comprised the full analysis set (FAS): triplet *n* = 111 and doublet *n* = 84. Four patients did not initiate therapy (Figure S1—see online supplementary material for a color version of this figure). Approximately 70% of the cohort had at least one prognostic factor, and treatment was given in second‑ or third‑line settings per protocol. Second‑line treatment accounted for the majority of patients in each cohort (triplet *n* = 94, doublet *n* = 68), with third‑line representing the remainder (triplet *n* = 17, doublet *n* = 16; Table 1).

**Table 1.** Patient characteristics.

	All (*n* = 195)	Triplet (*n* = 111)	Doublet (*n* = 84)
**Sex, *n* (%)**			
** Male/Female**	101 (51.8)/94 (48.2)	61 (55.0)/50 (45.0)	40 (47.6)/44 (52.4)
**Age, year**			
** median (range)**	67 (20 - 89)	64 (20 - 85)	71 (34 - 89)
**ECOG performance status, *n* (%)**			
** 0/1/2**	108 (55.4)/76 (39.0)/11 (5.6)	58 (52.3)/47 (42.3)/6 (5.4)	50 (59.5)/29 (34.5)/5 (6.0)
**Tumor sidedness, *n* (%)**			
** Right/Left/Unclassifiable**	91 (46.7)/94 (48.2)/10 (5.1)	55 (49.5)/52 (46.8)/4 (3.6)	36 (42.9)/42 (50.0)/6 (7.1)
**No. of metastatic sites, *n* (%)**			
** 0-2/3≤**	142 (72.8)/53 (27.2)	76 (68.5)/35 (31.5)	66 (78.6)/18 (21.4)
**Time to metastases, *n* (%)**			
** Synchronous/Metachronous**	128 (65.6)/67 (34.4)	80 (72.1)/31 (27.9)	48 (57.1)/36 (42.9)
**Ascites, *n* (%)**			
** Yes/No**	48 (24.6)/147 (75.4)	35 (31.5)/76 (68.5)	13 (15.5)/71 (84.5)
**MSI status, *n* (%)**			
** MSI-H/non-MSI-H/unknown**	12 (6.2)/159 (81.5)/24 (12.3)	6 (5.4)/87 (78.4)/18 (16.2)	6 (7.1)/72 (85.7)/6 (7.1)
**CRP, *n* (%)**			
** 1>/1≤/unknown**	131 (67.2)/61 (31.3)/3 (1.5)	64 (57.7)/45 (40.5)/2 (1.8)	67 (79.8)/16 (19.0)/1 (1.2)
**Prior regimens**			
** 1/2**	162 (83.1)/33 (16.9)	94 (84.7)/17 (15.3)	68 (81.0)/16 (19.0)
**Any prognostic factors** ^a^ **, *n* (%)**			
** Yes**	153 (78.5)	95 (85.6)	58 (69.0)

Abbreviations: ECOG, Eastern Cooperative Oncology Group; MSI, microsatellite instability.

a ECOG PS ≥ 1, number of metastatic organs ≥ 3, CRP level ≥ 1 mg/dL, and primary-tumor status unresected.

### Treatment exposure and dose intensity

By the time of data cutoff, treatment had been discontinued in 108 patients in the triplet cohort and in 76 patients in the doublet cohort. Among those patients, relative dose intensity for encorafenib and cetuximab was comparable: 75.4% vs 72.5% and 76.5% vs 78.9% for triplet and doublet cohort, respectively, indicating similar deliverability of the regimens in real‑world practice. The relative dose intensity for binimetinib was 53.9%.

### Clinical outcomes in each treatment cohort

At data cutoff, the median OS was 12.9 months (95% CI 10.7-15.2); the 12‑month OS rate was 52.6%. By line of therapy, survival curves showed broadly consistent outcomes between second‑ and third‑line strata. Median PFS was 4.9 months. Patterns by line of therapy mirrored the OS results, without overt separation of curves overall (Figures S2-S5—see online supplementary material for a color version of this figure). In the overall population, ORR and DCR were 39.0% (95%CI 31.7-46.2) and 80.2% (95%CI 74.3-86.2), median tumor shrinkage rate was 27.8% (range, -116.2-100), median TTR was 1.9 months (95%CI 1.8-2.0), and median DoR was 4.7 months (95%CI 3.7-6.0).

**Figure 1.**
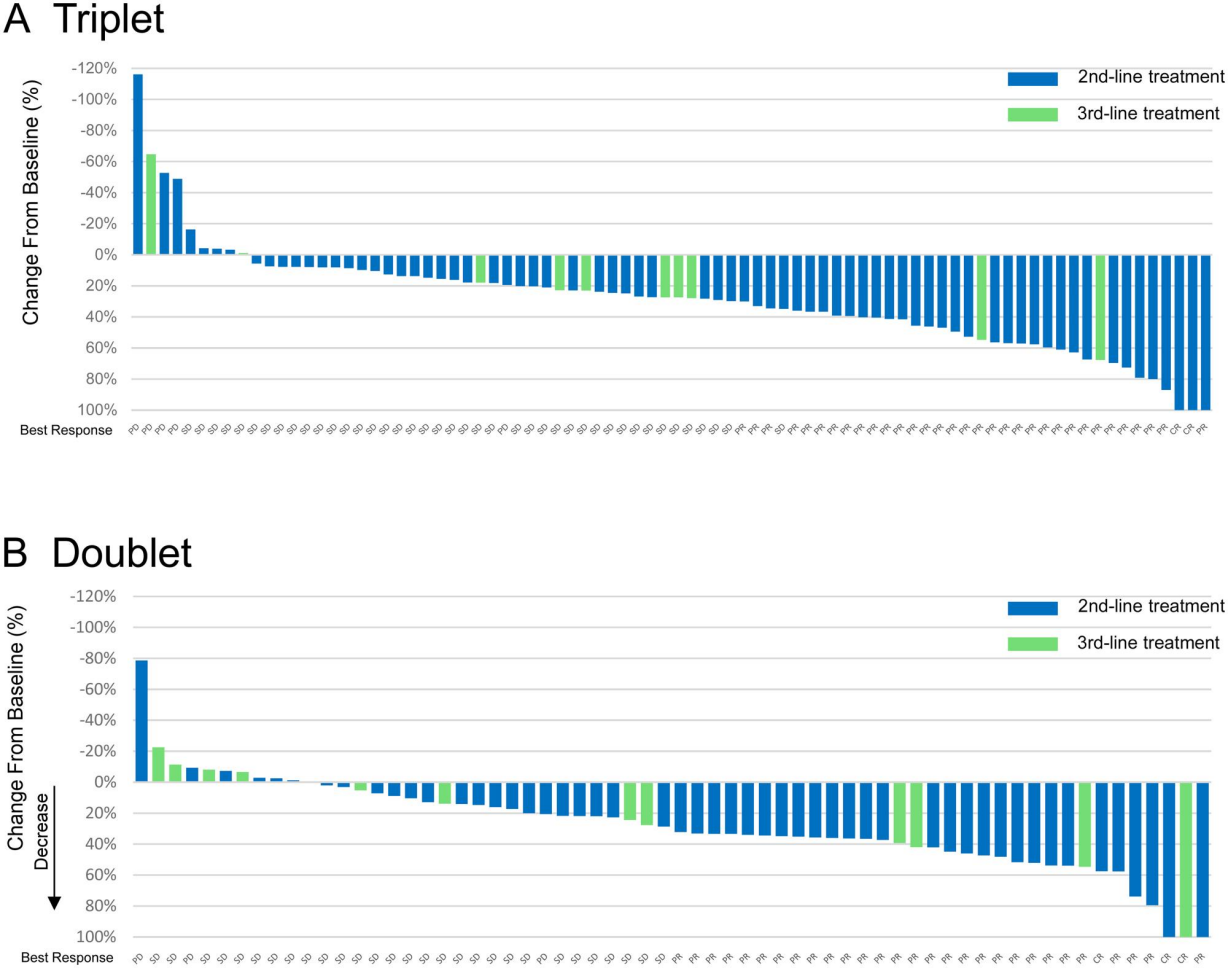
Waterfall plot of patients with measurable lesions in each treatment group. (A) triplet group, (B) doublet group.

Among patients with measurable disease at baseline, ORR was 35.0% and 44.4%, and DCR was 77.0% and 84.7%, in the triplet and doublet cohort, respectively (Tables S1 and S2—see online supplementary material). Waterfall plot showed favorable tumor shrinkage, consistent with prior reports from the BEACON trial (Figure 1).

### Comparison of triplet and doublet treatment

**Figure 2.**
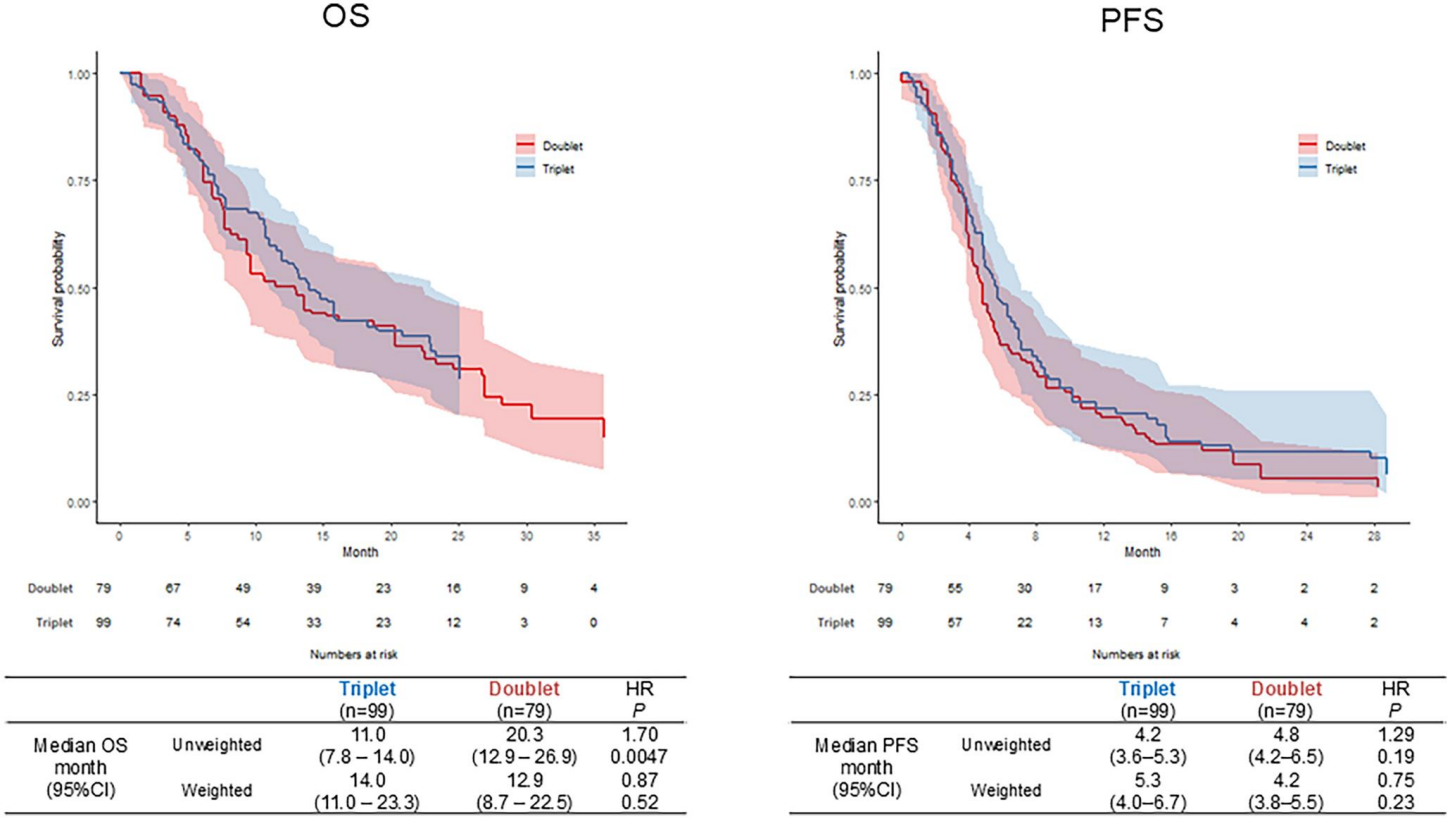
Comparison of survival time between triplet and doublet groups using inverse probability weighting analysis. Left: overall survival, right: progression-free survival.

In the evaluable patients, the doublet cohort was better in both OS and PFS compared to the triplet cohort (Figure S6—see online supplementary material for a color version of this figure). In the IPW analysis, median OS was 14.0 months (95%CI, 11.0-23.3) in the triplet cohort and 12.9 months (8.7-22.5) in the doublet cohort (HR 0.87, 95% CI 0.57-1.33, *P*-value .52). Median PFS was 5.3 months (4.0-6.7) for triplet versus 4.2 months (3.8-5.5) for doublet (HR 0.75, 95% CI 0.48-1.19, *P*-value .23; Figure 2). The ORR and DCR were comparable between the two cohorts (Table S3—see online supplementary material).

**Figure 3.**
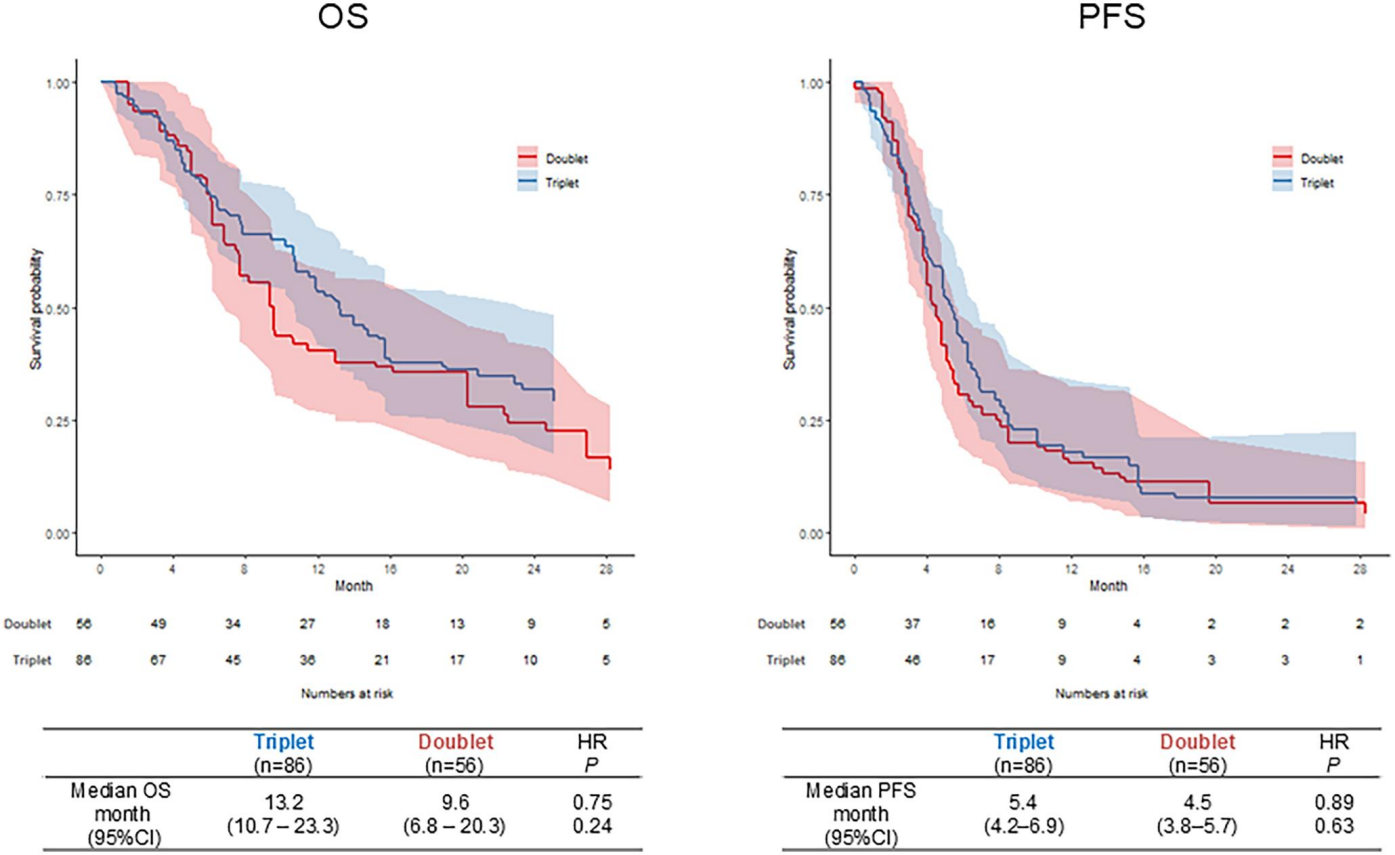
Comparison of survival time between triplet and doublet groups using inverse probability weighting analysis in patients with any prognostic factors. Left: overall survival, right: progression-free survival.

In accordance with the Japanese Colorectal Cancer treatment guidelines recommendation, triplet therapy is preferentially administered to patients with poor prognostic factors. Therefore, we performed IPW-based comparison among patients with at least one prognostic factor (*n* = 142 after IPW). In this subset of patients, the triplet cohort demonstrated numerically longer OS (13.2 vs 9.6 months; HR 0.75, 95% CI 0.46-1.22, *P*-value .24) and a modest trend toward longer PFS (5.4 vs 4.5 months; HR 0.89, 95% CI 0.54-1.45, *P*-value .52) compared with the doublet cohort (Figure 3). The ORR and DCR were comparable between the two cohorts, although it was numerically higher in the doublet cohort (Table S3—see online supplementary material).

### Safety

Adverse events were manageable and consistent with known profiles of encorafenib/cetuximab ± binimetinib. The most frequent toxicities included fatigue, acneiform rash, diarrhea, and anemia. Grade ≥ 3 events occurred in roughly a quarter of patients in each cohort, without new safety signals or treatment‑related deaths (Table 2).

**Table 2.** Safety profile.

	All		Triplet		Doublet	
	All grades, *n* (%）	Grade ≥ 3, *n* (%）	All Grades, *n* (%）	Grade ≥ 3, *n* (%）	All Grades, *n* (%）	Grade ≥ 3, *n* (%）
**White blood cell decreased**	19 (9.7)	2 (1.0)	13 (11.7)	2 (1.8)	6 (7.1)	0 (0.0)
**Neutrophil count decreased**	23 (11.8)	1 (0.5)	15 (13.5)	0 (0.0)	8 (9.5)	1 (1.2)
**Platelet count decreased**	34 (17.4)	2 (1.0)	15 (13.5)	1 (0.9)	19 (22.6)	1 (1.2)
**Anemia**	89 (45.6)	32 (16.4)	58 (52.3)	25 (22.5)	31 (36.9)	7 (8.3)
**Aspartate aminotransferase increased**	81 (41.5)	6 (3.1)	52 (46.8)	4 (3.6)	29 (34.5)	2 (2.4)
**Alanine aminotransferase increased**	83 (42.6)	4 (2.1)	44 (39.6)	2 (1.8)	39 (46.4)	2 (2.4)
**Creatinine increased**	65 (33.3)	3 (1.5)	52 (46.8)	3 (2.7)	13 (15.5)	0 (0.0)
**Diarrhea**	64 (32.8)	4 (2.1)	49 (44.1)	3 (2.7)	15 (17.9)	1 (1.2)
**Nausea**	81 (41.5)	12 (6.2)	57 (51.4)	9 (8.1)	24 (28.6)	3 (3.6)
**Vomiting**	40 (20.5)	4 (2.1)	31 (27.9)	2 (1.8)	9 (10.7)	2 (2.4)
**Mucositis oral**	31 (15.9)	2 (1.0)	20 (18.0)	2 (1.8)	11 (13.1)	0 (0.0)
**Fatigue**	60 (30.8)	10 (5.1)	39 (35.1)	9 (8.1)	21 (25.0)	1 (1.2)
**Malaise**	101 (51.8)	11 (5.6)	61 (55.0)	9 (8.1)	40 (47.6)	2 (2.4)
**Anorexia**	105 (53.8)	11 (5.6)	63 (56.8)	8 (7.2)	42 (50.0)	3 (3.6)
**Myalgia**	24 (12.3)	3 (1.5)	15 (13.5)	2 (1.8)	9 (10.7)	1 (1.2)
**Rash acneiform**	109 (55.9)	10 (5.1)	64 (57.7)	9 (8.1)	45 (53.6)	1 (1.2)
**Dry skin**	78 (40)	3 (1.5)	48 (43.2)	3 (2.7)	30 (35.7)	0 (0.0)
**Blurred vision**	26 (13.3)	0 (0.0)	18 (16.2)	0 (0.0)	8 (9.5)	0 (0.0)
**Paronychia**	29 (14.9)	3 (1.5)	18 (16.2)	2 (1.8)	11 (13.1)	1 (1.2)

## Discussion

This nationwide, prospective, real‑world study provides contemporary effectiveness and safety data for encorafenib plus cetuximab with or without binimetinib in *BRAF*‑mutated mCRC across 66 Japanese institutions. Median OS (∼13 months) and PFS (∼5 months) closely mirror outcomes observed in the BEACON CRC trial and are broadly concordant with Western real‑world cohorts, supporting generalizability of the BEACON findings to daily practice in Asian populations.^3^^,^^4^ In Japan, both triplet and doublet regimens are available in routine clinical practice. In the present study, the triplet cohort included a higher proportion of patients with advanced disease features. This likely reflects treatment selection patterns in real-world practice, whereby triplet therapy is preferentially administered to patients with poor prognostic factors in accordance with recommendations from the Japanese Society for Cancer of the Colon and Rectum.^5^ Consequently, the shorter survival observed in the triplet cohort is likely due to imbalances in baseline patient characteristics.

Although this study did not predefine a comparison between the doublet and triplet cohorts, we conducted exploratory analyses to generate evidence that may inform regimen selection in clinical practice. To account for imbalances in baseline characteristics between the two cohorts, IPW was applied. In this adjusted analysis, no significant differences in survival outcomes were observed between the cohorts, consistent with the findings from the subgroup analyses of the BEACON trial.^3^ Nonetheless, since triplet therapy is usually reserved for patients with poor prognostic factors, we further explored this clinical question and performed an additional analysis restricted to patients with adverse prognostic features. Although the difference did not reach statistical significance, OS tended to be more favorable in the triplet cohort. These results are directionally aligned with subgroup observations from the BEACON and with Japan‑specific recommendations that emphasize triplet therapy in patients with higher tumor burden or inflammation.

The safety profiling in our study was consistent with the established toxicity spectrum of the BEACON trial,^4^ with no new signals. This is clinically important in a population enriched for advanced disease and prior therapies. Real‑world dose intensity of encorafenib and cetuximab was comparable across arms; however, the dose intensity of binimetinib (53.9%) was lower than that in the BEACON trial (87%). The higher proportion of patients with poor prognostic factors in the triplet cohort, together with toxicity management through dose reductions in real-world clinical practice, may have influenced the outcomes. The median PFS in the overall cohort was 4.9 months, which is consistent with the results of the BEACON trial. No apparent association was observed between dose intensity and treatment efficacy.

Recently, treatment paradigms for *BRAF* V600E-mutated pMMR/MSS mCRC have rapidly evolved. The phase III BREAKWATER trial demonstrated a significant improvement in ORR and PFS with encorafenib plus cetuximab combined with chemotherapy compared with standard chemotherapy alone in the first-line setting, supporting the emergence of chemotherapy plus encorafenib and cetuximab as a new standard of care.^9^ In parallel, the phase II ANCHOR CRC study showed clinically meaningful activity of encorafenib, binimetinib, and cetuximab in previously untreated patients, although the durability of benefit remained limited.^10^ In our study, although the addition of binimetinib did not result in a statistically significant survival advantage over doublet therapy in the overall population, exploratory analyses suggested a numerical benefit of triplet therapy in patients with adverse prognostic features. This observation is consistent with the biological rationale derived from ANCHOR and BEACON, in which more intensive MAPK pathway inhibition may be required to overcome aggressive tumor biology. As encorafenib plus cetuximab in combination with chemotherapy becomes increasingly adopted in the first-line setting, the identification of patient subsets who may benefit from subsequent triplet therapy will become an important clinical question.

Our study has several limitations regarding study design and analysis methods. Although comparing 2 cohorts for the efficacy, our study was a nonrandomized design. Residual confounding may persist despite IPW analysis, and the study was not powered for definitive comparative effectiveness between triplet and doublet therapy. However, these results would support both regimens as effective standard options for previously treated *BRAF*‑mutated mCRC in practice, with a potential clinical edge for triplet therapy in patients harboring poor prognostic features. Pending biomarker results from our study may refine treatment selection further—e.g., prioritizing triplet therapy in BM1‑like, high‑MAPK signaling tumors or in RNF43‑altered disease, and reserving doublet therapy for patients with competing frailty considerations.^11–14^

## Conclusion

In this nationwide prospective real‑world study, encorafenib plus cetuximab with or without binimetinib achieved survival and response outcomes comparable to the BEACON CRC trial, with manageable safety. Triplet therapy may be preferential in patients with poor prognostic features. Ongoing paired liquid‑biopsy analyses are expected to clarify molecular determinants of benefit and resistance, enabling more precise personalization of therapy in *BRAF*‑mutated mCRC.

## Supplementary Material

oyag068_Supplementary_Data

## Data Availability

The dataset analyzed during the current study are available from the corresponding author on reasonable request (jaccro@jaccro.or.jp).
