# Supplementary material for: Real-world outcomes of encorafenib, cetuximab ± binimetinib for BRAF‑mutated metastatic colorectal cancer: the BEETS (JACCRO CC‑18) study
Source: Oncologist. 2026 Feb 27;31(4):oyag068. doi: 10.1093/oncolo/oyag068 (PMC13006056; doi:10.1093/oncolo/oyag068)
Supplement: oyag068_Supplementary_Data [file oyag068_supplementary_data.zip › Supplementary Table 3.docx]

**Supplementary Table 3. Tumor response between triplet and doublet cohort by IPW analysis in patients with measurable lesions and prognostic factors**

|  | Triplet | Doublet | *P* |
| --- | --- | --- | --- |
| Patients with measurable lesions | | | |
| n | 99 | 79 |  |
| Objective response rate |  |  |  |
| %  (95%CI) | 37.4  (25.2 – 49.6) | 46.4  (32.0 – 60.8) | 0.35 |
| Disease control rate |  |  |  |
| %  (95%CI) | 77.4  (67.7 – 87.3) | 84.9  (75.3 – 94.5) | 0.29 |
| Patients with measurable lesions and prognostic factors | | | |
| n | 86 | 56 |  |
| Objective response rate |  |  |  |
| %  (95%CI) | 35.6  (21.9 – 49.4) | 42.3  (24.7 – 60.2) | 0.55 |
| Disease control rate |  |  |  |
| %  (95%CI) | 76.0  (64.9 – 87.0) | 87.2  (77.4 – 96.9) | 0.14 |
