# Supplementary material for: Real-world outcomes of encorafenib, cetuximab ± binimetinib for BRAF‑mutated metastatic colorectal cancer: the BEETS (JACCRO CC‑18) study
Source: Oncologist. 2026 Feb 27;31(4):oyag068. doi: 10.1093/oncolo/oyag068 (PMC13006056; doi:10.1093/oncolo/oyag068)
Supplement: oyag068_Supplementary_Data [file oyag068_supplementary_data.zip › Supplementary Table 1.docx]

**Supplementary Table 1. Tumor response in the triplet cohort in patients with measurable lesions**

|  | Triplet, all  (n=100) | 2nd-line  (n=85) | 3rd-line  (n=15) | *P* |
| --- | --- | --- | --- | --- |
| CR, n (%) | 2 (2.0) | 2 (2.4) | 0 (0.0) |  |
| PR, n (%) | 33 (33.0) | 31 (36.5) | 2 (13.3) |  |
| SD, n (%) | 42 (42.0) | 35 (41.2) | 7 (46.7) |  |
| PD, n (%) | 5 (5.0) | 4 (4.7) | 1 (6.7) |  |
| NE, n (%) | 18 (18.0) | 13 (15.3) | 5 (33.3) |  |
| ORR, % (95%CI) | 35.0 (25.7 – 44.3) | 38.8 (28.5 – 49.2) | 13.3 (0.0 – 30.5) | 0.056 |
| DCR, % (95%CI) | 77.0 (68.8 – 85.2) | 80.0 (71.5 – 88.5) | 60.0 (35.2 – 84.8) | 0.090 |
