# Supplementary material for: Real-world outcomes of encorafenib, cetuximab ± binimetinib for BRAF‑mutated metastatic colorectal cancer: the BEETS (JACCRO CC‑18) study
Source: Oncologist. 2026 Feb 27;31(4):oyag068. doi: 10.1093/oncolo/oyag068 (PMC13006056; doi:10.1093/oncolo/oyag068)
Supplement: oyag068_Supplementary_Data [file oyag068_supplementary_data.zip › Supplementary Table 2.docx]

**Supplementary Table 2. Tumor response in the doublet cohort in patients with measurable lesions**

|  | Doublet, all  (n=72) | 2nd-line  (n=59) | 3rd-line  (n=13) | *P* |
| --- | --- | --- | --- | --- |
| CR, n (%) | 3 (4.2) | 2 (3.4) | 1 (7.7) |  |
| PR, n (%) | 29 (40.3) | 26 (44.1) | 3 (23.1) |  |
| SD, n (%) | 29 (40.3) | 21 (35.6) | 8 (61.5) |  |
| PD, n (%) | 3 (4.2) | 3 (5.1) | 0 (0.0) |  |
| NE, n (%) | 8 (11.1) | 7 (11.9) | 1 (7.7) |  |
| ORR, % (95%CI) | 44.4 (33.0 – 55.9) | 47.5 (34.7 – 60.2) | 30.8 (5.7 – 55.9) | 0.27 |
| DCR, % (95%CI) | 84.7 (76.4 – 93.0) | 83.1 (73.5 – 92.6) | 92.3 (77.8 – 100.0) | 0.40 |
