# Supplementary figures and images for: Real-world outcomes of encorafenib, cetuximab ± binimetinib for BRAF‑mutated metastatic colorectal cancer: the BEETS (JACCRO CC‑18) study
Source: Oncologist. 2026 Feb 27;31(4):oyag068. doi: 10.1093/oncolo/oyag068 (PMC13006056; doi:10.1093/oncolo/oyag068)

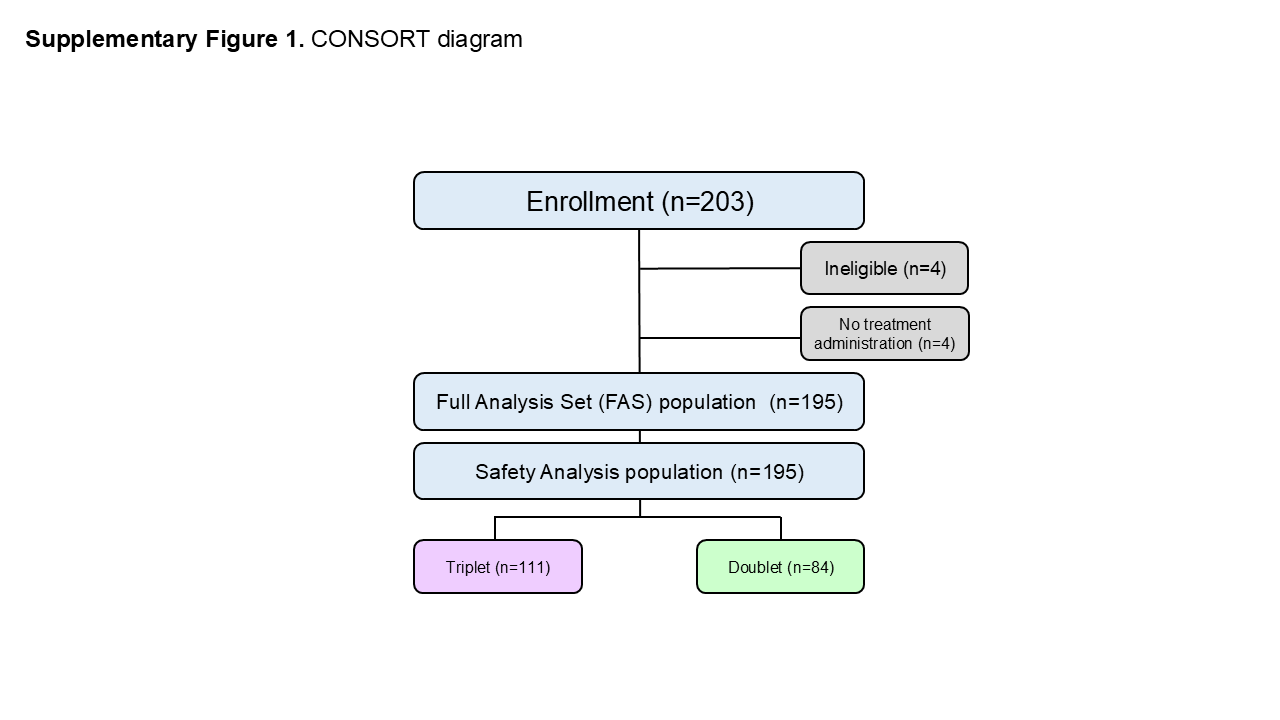

Supplement: oyag068_Supplementary_Data [file oyag068_supplementary_data.zip › Supplementary Figure 1.tif]

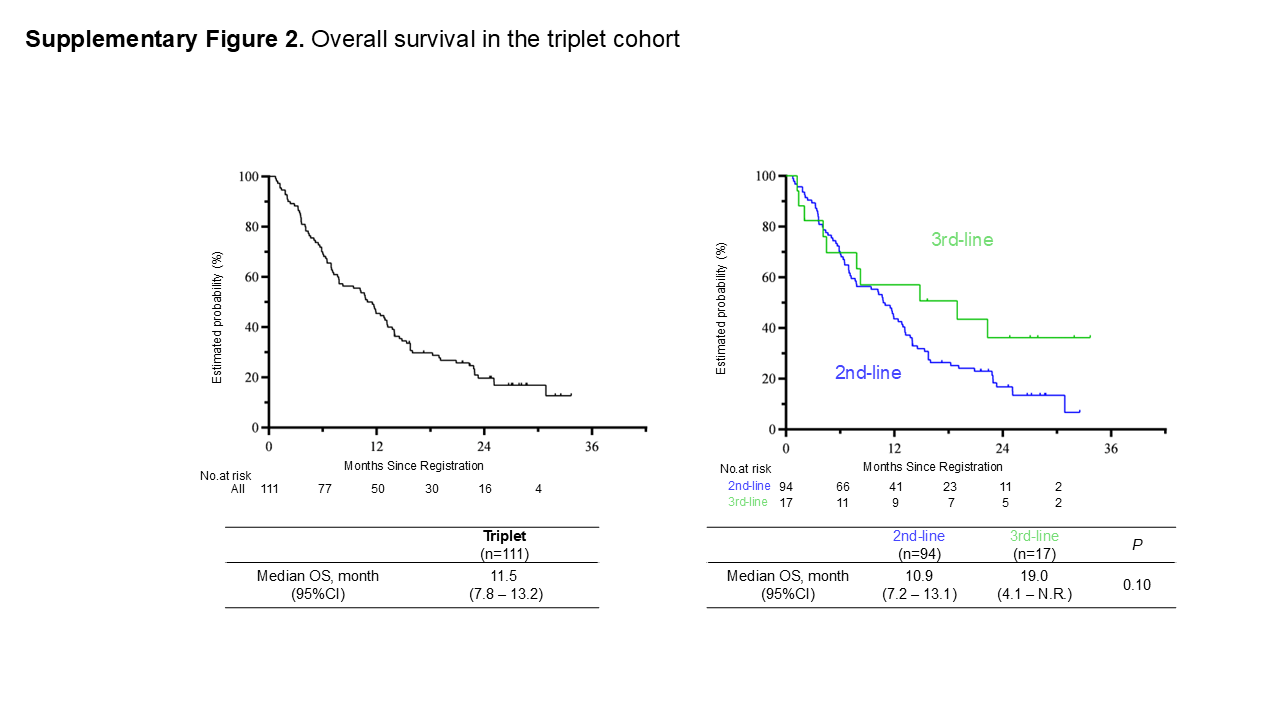

Supplement: oyag068_Supplementary_Data [file oyag068_supplementary_data.zip › Supplementary Figure 2.tif]

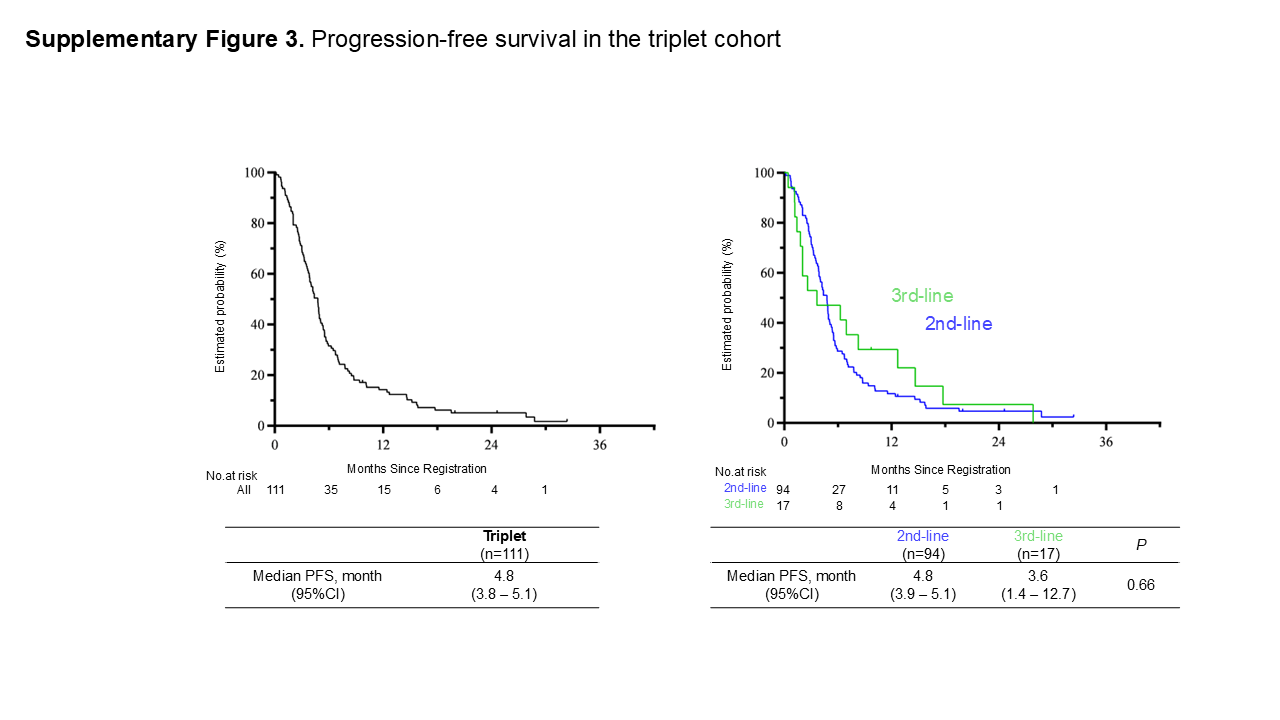

Supplement: oyag068_Supplementary_Data [file oyag068_supplementary_data.zip › Supplementary Figure 3.tif]

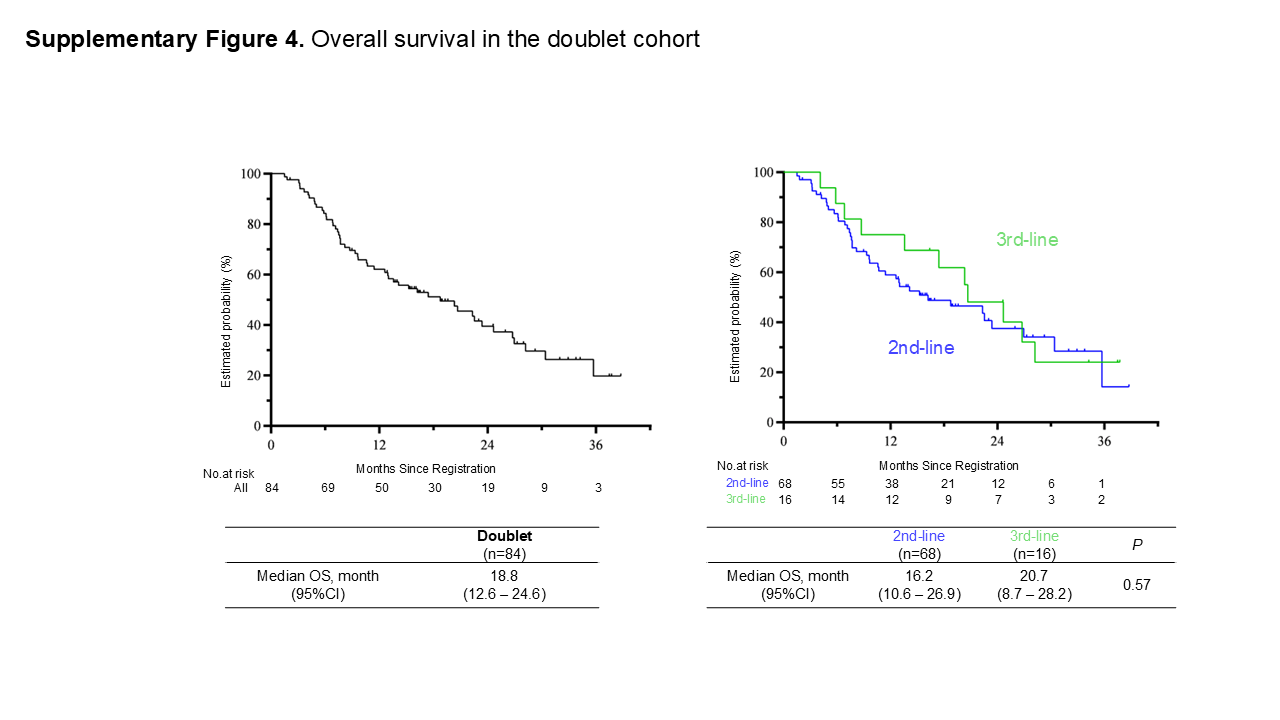

Supplement: oyag068_Supplementary_Data [file oyag068_supplementary_data.zip › Supplementary Figure 4.tif]

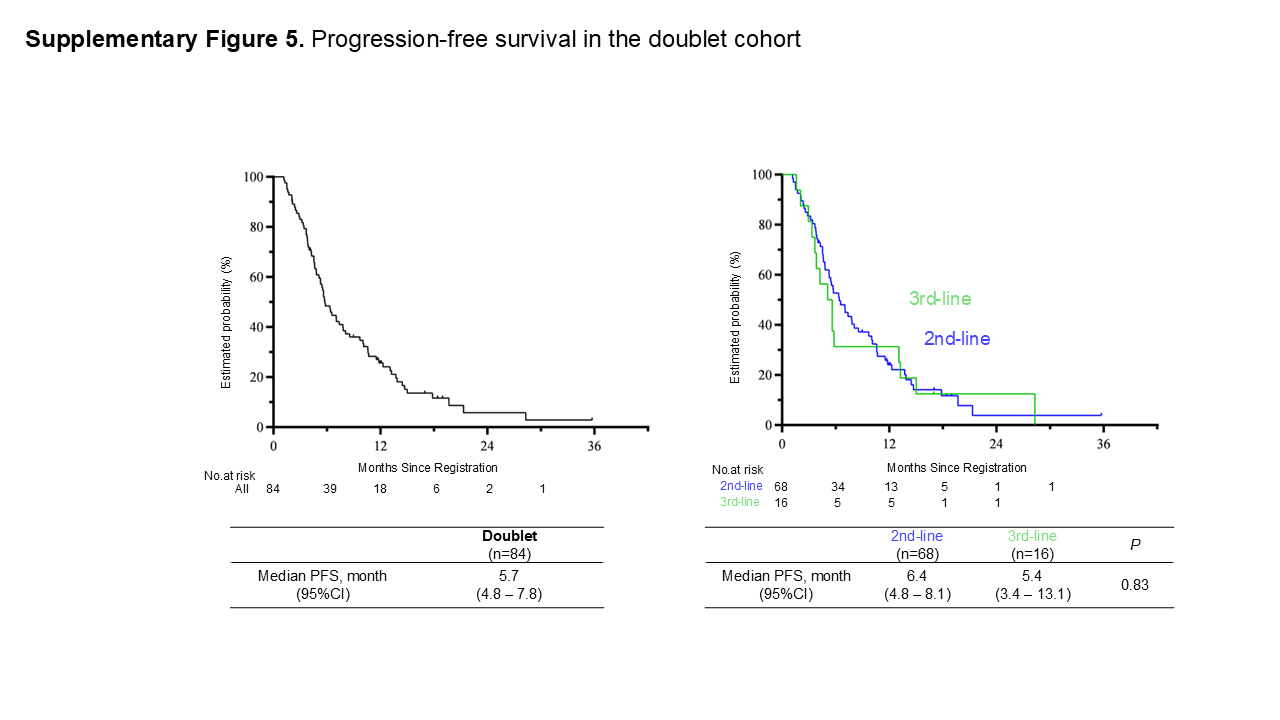

Supplement: oyag068_Supplementary_Data [file oyag068_supplementary_data.zip › Supplementary Figure 5.tif]

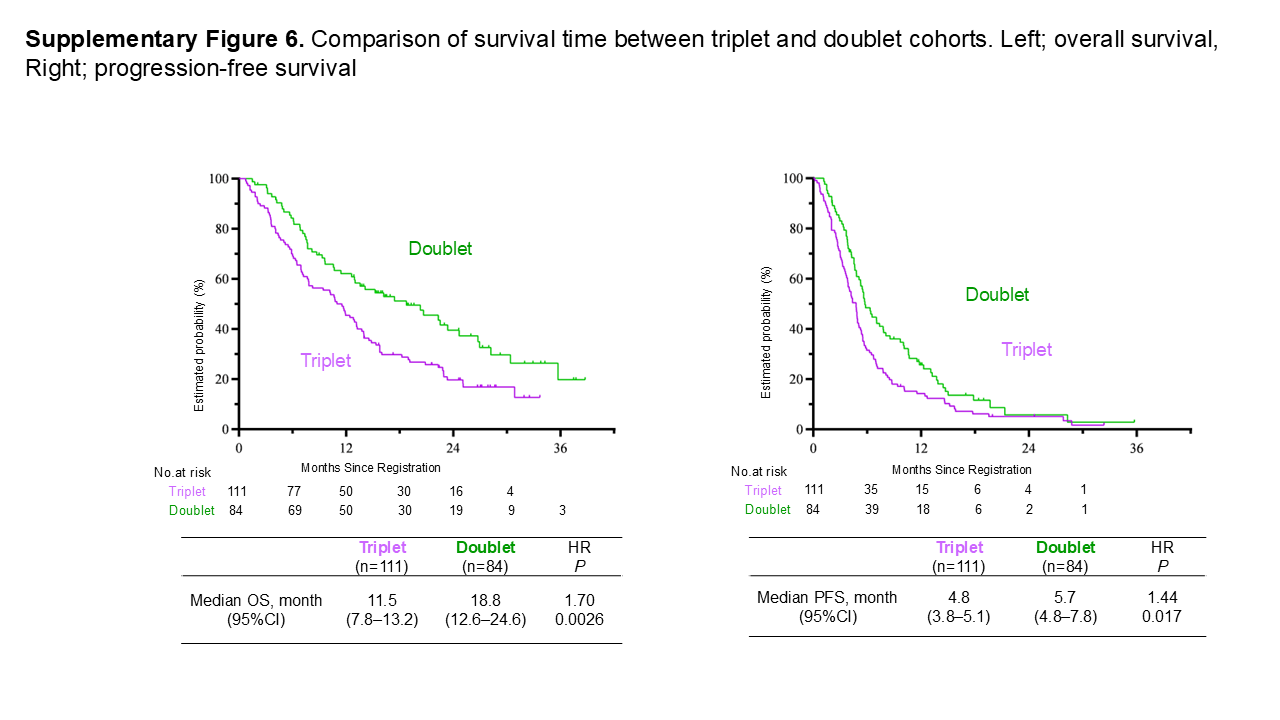

Supplement: oyag068_Supplementary_Data [file oyag068_supplementary_data.zip › Supplementary Figure 6.tif]
